# Synthesis, crystal structure and Hirshfeld surface analysis of *N*-(2,6-di­methyl­phen­yl)-2-morpholinoacetamide, a Lidocaine analog

**DOI:** 10.1107/S2056989026006043

**Published:** 2026-06-16

**Authors:** Imane Maimoune, Abderrazzak El Moutaouakil Ala Allah, Benson M. Kariuki, Abdulsalam Alsubari, Joel T. Mague, Abdelkader Zarrouk, Youssef Ramli

**Affiliations:** a Laboratory of Medicinal Chemistry Drug Sciences Research Center Faculty of Medicine and Pharmacy Mohammed V University in Rabat Morocco; b Laboratory of Materials Nanotechnology and Environment Faculty of Sciences Mohammed V University in Rabat PO Box 1014 Rabat Morocco; c School of Chemistry, Cardiff University, Main Building Park Place, Cardiff, CF10 3AT, United Kingdom; d Laboratory of Medicinal Chemistry, Faculty of Clinical Pharmacy, 21 September University, Yemen; e Department of Chemistry Tulane University New Orleans LA 70118 USA; Universidade de Sâo Paulo, Brazil

**Keywords:** crystal structure, acetamide, hydrogen bond, morpholine, Lidocaine, Hirshfeld surface

## Abstract

In the title mol­ecule, C_14_H_20_N_2_O_2_, the dihedral angle between the mean plane of the phenyl ring and that defined by the *ipso*-C—NH—(C= O)—CH_2_— unit is 66.59 (11)°. The morpholine unit adopts a chair conformation. In the crystal, N—H⋯O hydrogen bonds and C—H⋯π(ring) inter­actions form chains extending along the *b*-axis direction. A Hirshfeld surface analysis showed H⋯H contacts to constitute nearly 70% of the inter­molecular contacts in the crystal.

## Abstract 2

In the title mol­ecule, the dihedral angle between the mean plane of the phenyl ring and that defined by the *ipso*-C—NH—(C=O)—CH_2_— unit is 66.59 (11)°. In the crystal, N—H⋯O hydrogen bonds and C—H⋯π(ring) inter­actions form chains extending along the *b*-axis direction. A Hirshfeld surface analysis showed H⋯H contacts to constitute nearly 70% of the inter­molecular contacts in the crystal.

## 1. Chemical context

In medicinal chemistry, heterocyclic compounds, particularly those with a nitro­gen atom, are crucial, forming the core of over 90% of new drugs and vital biomolecules such as vitamins and DNA (Al Mulla, 2017 ▸). Numerous studies on the acetamide family have shown that it can be found in a variety of well-known medications from different classes with a range of therapeutic effects. They have a wide range of biological activities due to their structural resemblance to numerous bioactive natural and synthetic mol­ecules (Missioui *et al.*, 2022*a* ▸). A heterocyclic substance with local anesthetic properties is lidocaine. It is composed of a hydro­philic amine and a lipophilic aromatic ring. Its primary target in excitable cells is the voltage-gated sodium channel, which causes the elevated sodium permeability seen in skeletal muscles, peripheral nerves, neuroendocrine, and cardiac cells during the rising phase of the action potential.
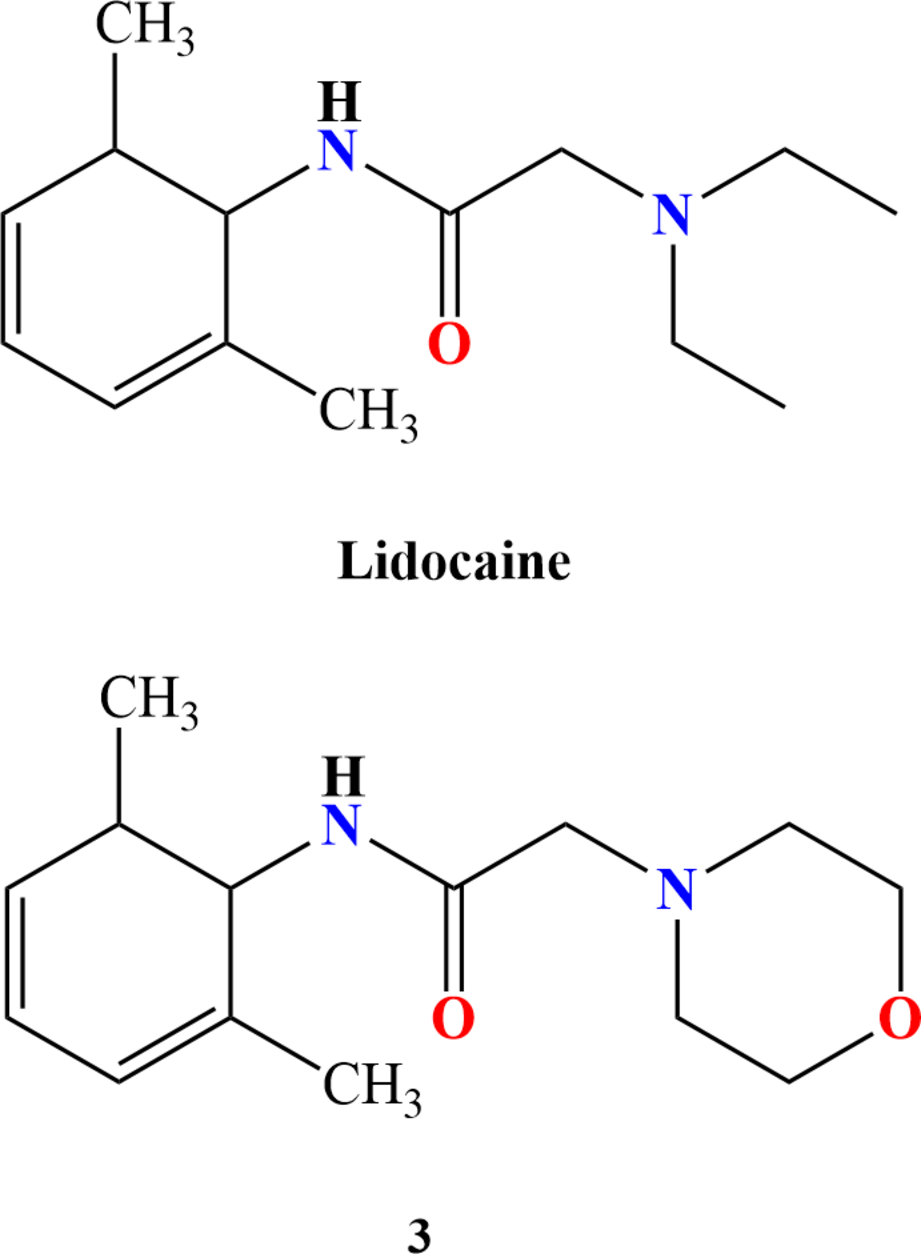


As part of our research in this field (Maimoune *et al.*, 2025 ▸), we synthesized the lidocaine analogue *N*-(2,6-di­methyl­phen­yl)-2-morpholino­acetamide, **3**, via an alkyl­ation reaction of morpholine by 2-chloro-*N*-(2,6-di­methyl­phen­yl)acetamide under refluxing toluene, the crystal structure of which is presented in this paper. The inter­molecular inter­actions were examined using a Hirshfeld surface analysis.

## 2. Structural commentary

**Figure 1:**
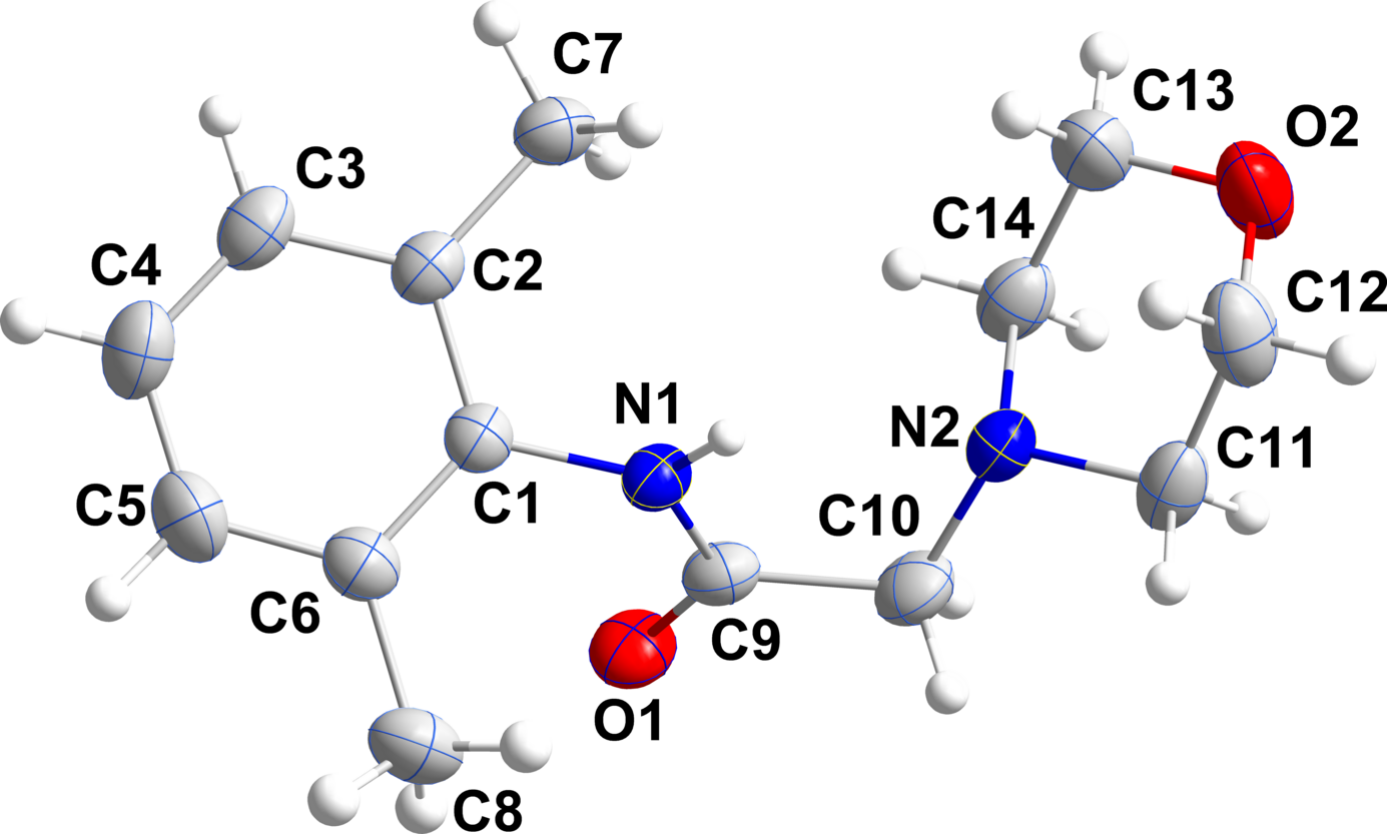
Perspective view of the title mol­ecule with the abeling scheme and 30% probability ellipsoids.

In the title mol­ecule **3**, Fig. 1 ▸, the dihedral angle between the mean plane of the C1–C6 ring and the plane defined by atoms C1/N1/C9/C10 is 66.59 (11)° while the dihedral angle between the latter plane and that defined by atoms C11–C14 is 63.59 (11)°. The morpholine unit adopts a chair conformation. Bond lengths and inter­bond angles are as expected. An intra­molecular N—H⋯N contact is observed (Table 1 ▸).

## 3. Supra­molecular features

**Figure 2:**
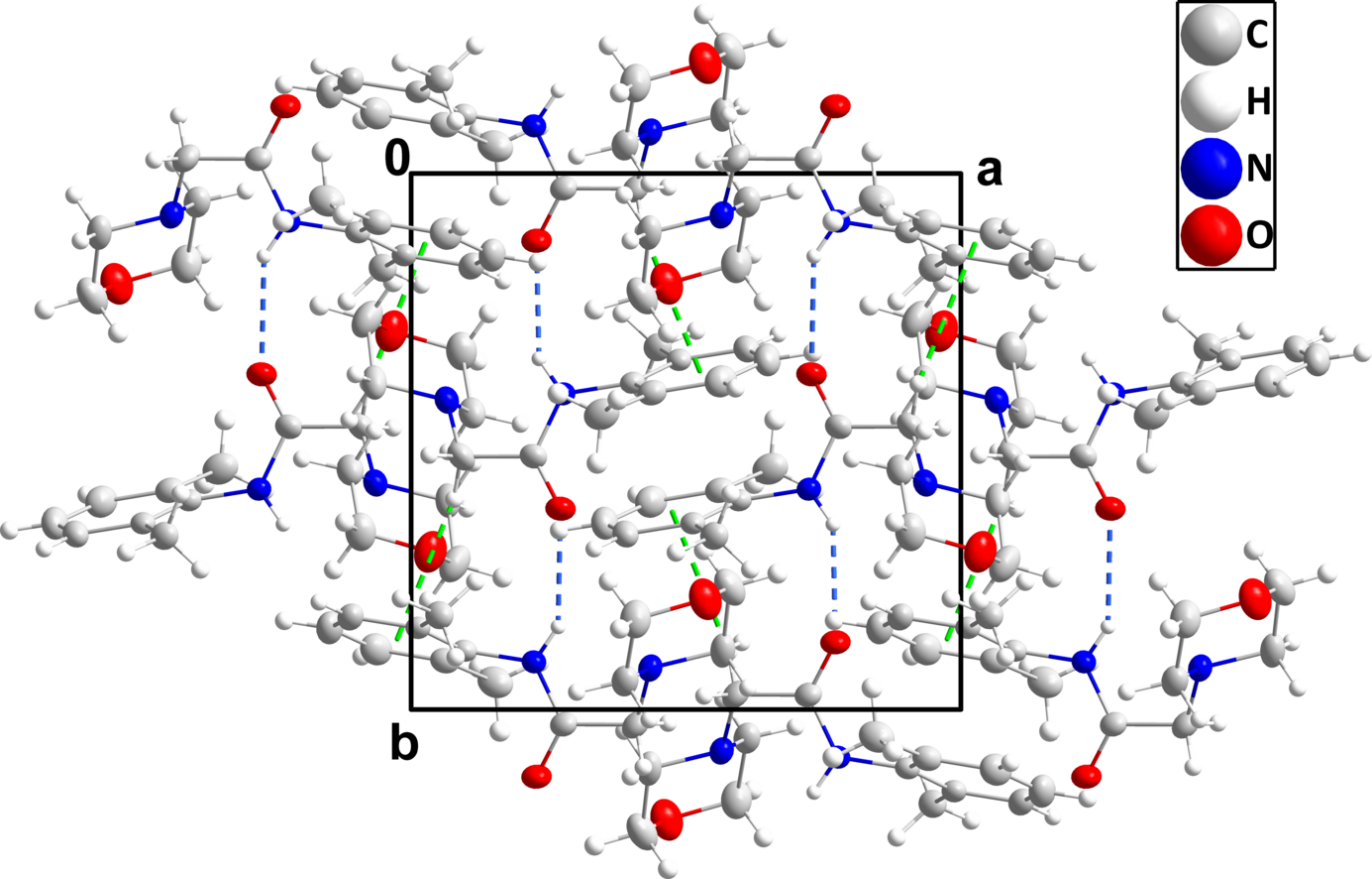
Packing viewed along the *c*-axis direction with N—H⋯O hydrogen bonds and C—H⋯π(ring) inter­actions depicted, respectively, by blue and green dashed lines.

In the crystal, N1—H1⋯O1^i^ hydrogen bonds and C10—H10*B*⋯*Cg*2^ii^ inter­actions (Table 1 ▸) form chains extending along the *b*-axis direction (Fig. 2 ▸). The chains largely pack with normal van der Waals contacts.

## 4. Database survey

**Figure 3:**
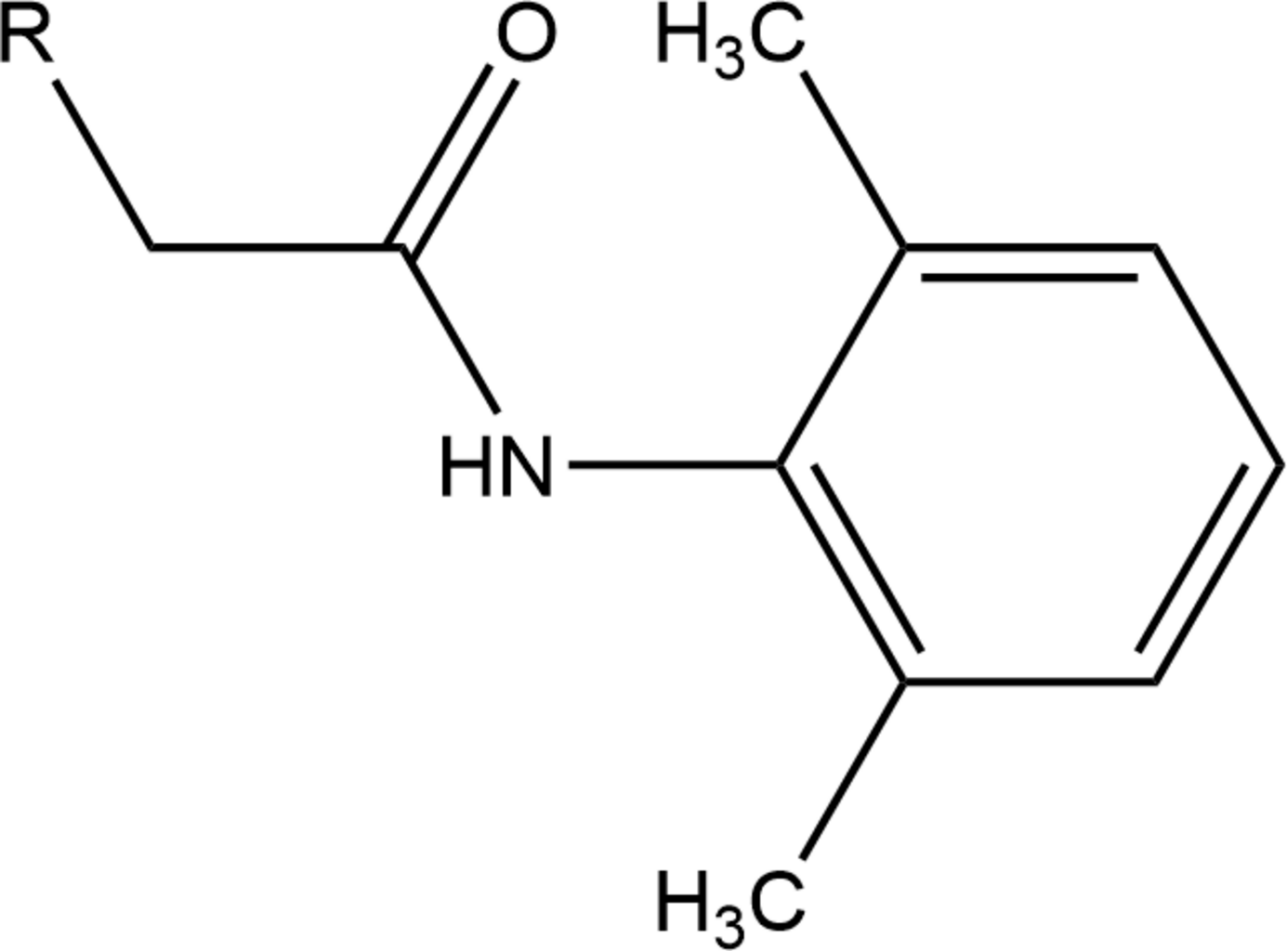
The search fragment used for the *Database survey*.

A search of the Cambridge Structural Database (CSD, updated to April 2026; Groom, *et al.*, 2016 ▸) with the fragment pictured in Fig. 3 ▸ (*R* = N) gave 99 hits, many of which were either metal complexes or salts in which *R* = *R*′*R*′′NH^+^ (*R*′ and *R*′′ = alkyl groups). Excluding these, 36 hits remained that were considered similar to the title mol­ecule and of these, 25 were co-crystals (Table 2 ▸ with *R* defined in Fig. 3 ▸). One of the more salient qu­anti­ties common to all, and the most likely to vary, is the dihedral angle between the mean plane of the 2,6-di­methyl­phenyl ring and the plane defined by the *ipso*-C—NH—(C=O)—CH_2_— unit. This is likely to be large to avoid close contacts between the methyl and carbonyl groups. Indeed, the smallest value is 57.2 (2)° in CINBEK but can be as large as 86.4 (3)° in WEDXAH, although the majority are in the range 60–75° as is the case for **3**. Considering those that are not co-crystals, this angle has a range of 57.2 (2)° (in CINBEK) to 82.0° (in LIDCAN10) and since the *R* group is fairly remote from the *ipso*-C—N bond, the variation is likely due to packing considerations. A comparable range for the dihedral angle is also seen in the co-crystals and there does not appear to be any definite correlation with the size of the second component.

## 5. Hirshfeld surface analysis

**Figure 4:**
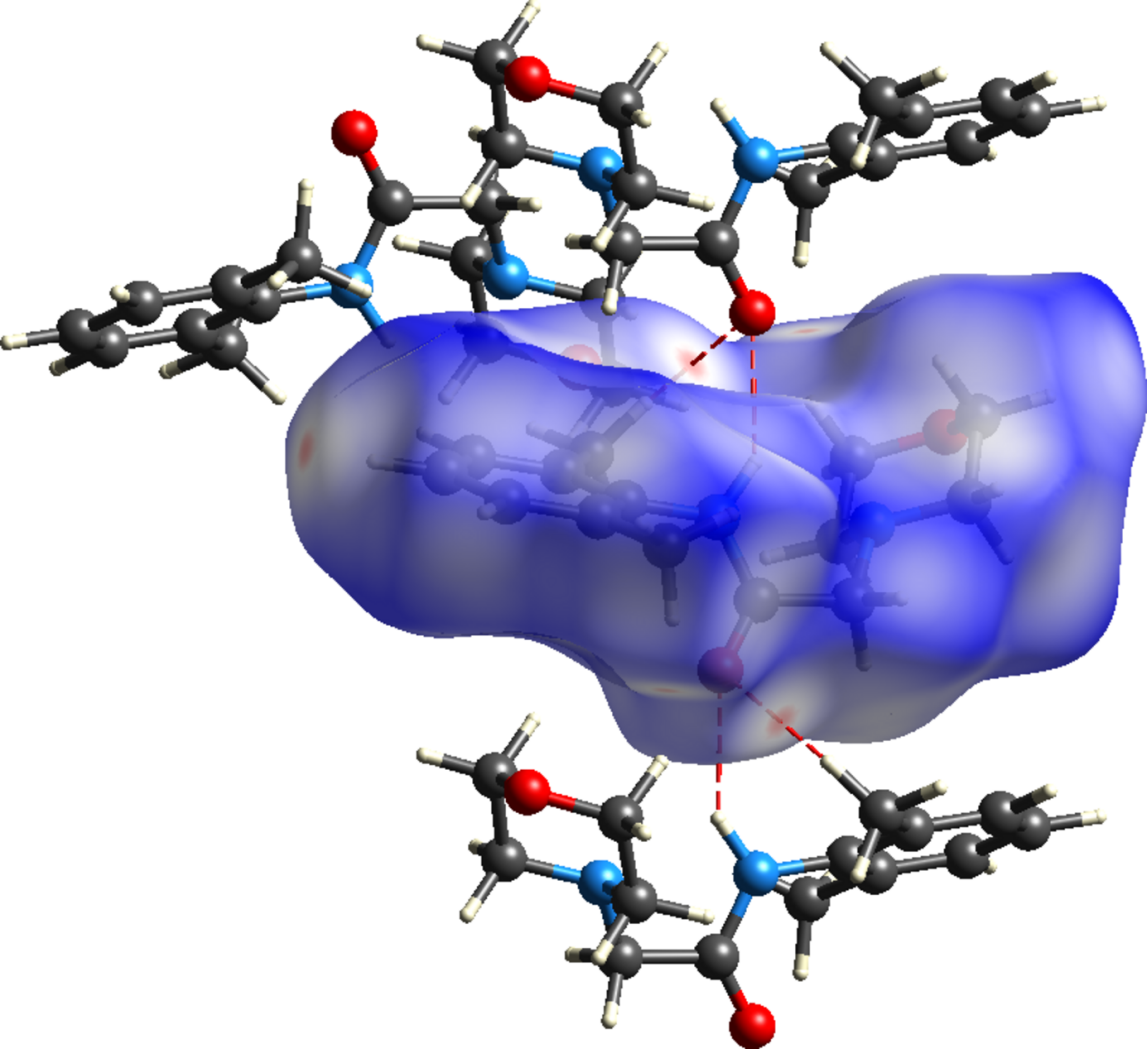
The *d*_norm_ Hirshfeld surface of the title mol­ecule with two neighboring mol­ecules in the hydrogen-bonded chain. The N—H⋯O hydrogen bonds are shown as dashed lines.

**Figure 5:**
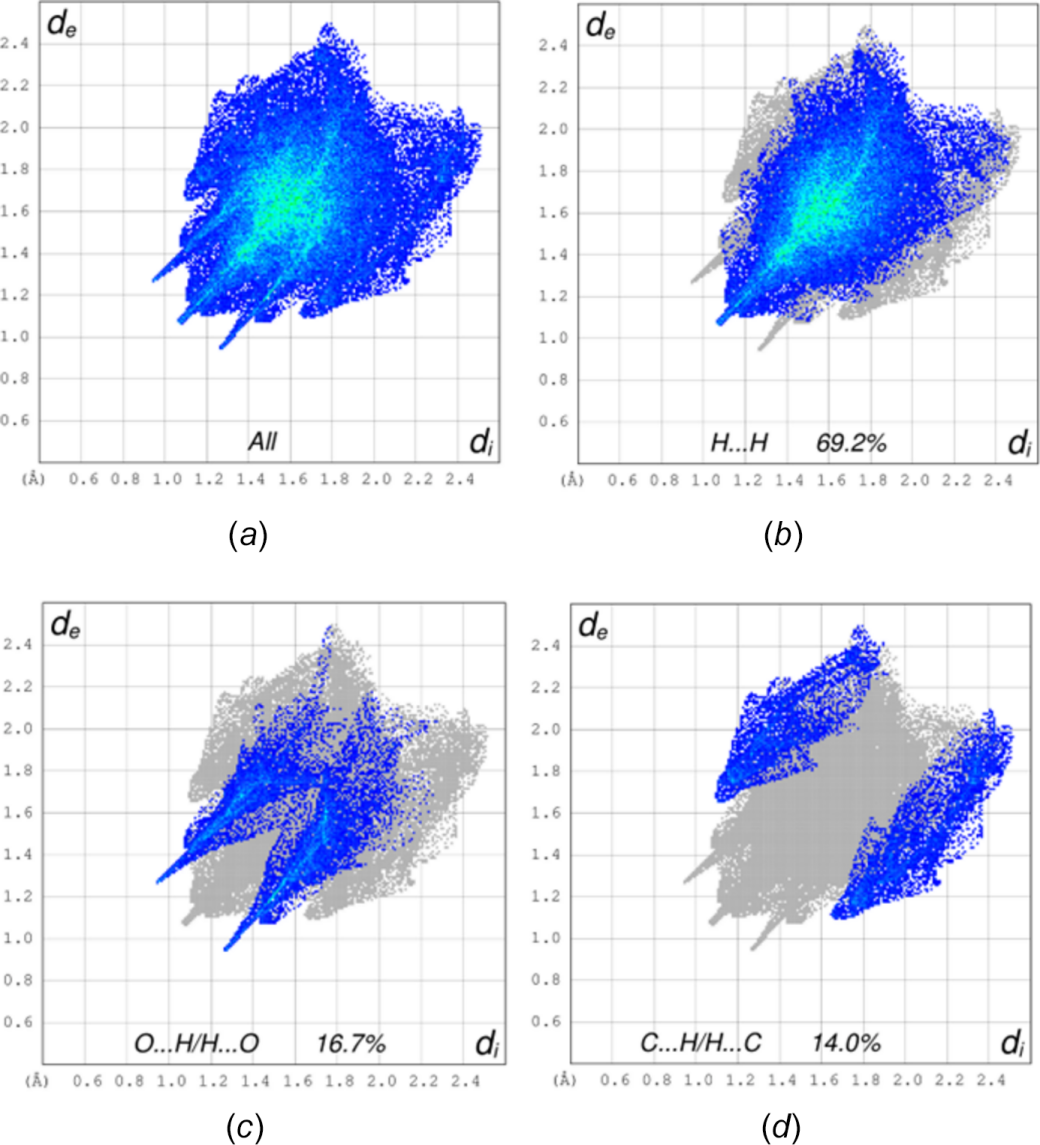
The two-dimensional fingerprint plots for the title mol­ecule showing (*a*) all contacts, (*b*) H⋯H contacts, (*c*) O⋯H/H⋯O contacts and (*d*) C⋯H/H⋯C contacts.

The Hirshfeld surface of **3** was calculated with *CrystalExplorer17* (Spackman *et al.*, 2021 ▸) and mapped over *d*_norm_ from −0.1546 to 1.1576 in arbitrary units. It is shown, together with two neighboring mol­ecules and the hydrogen bonds between them, in Fig. 4 ▸. Details of the appearance and inter­pretations of the plots generated by *CrystalExplorer* have been published (Tan *et al.*, 2019 ▸). The ensemble in Fig. 4 ▸ is a portion of the hydrogen-bonded chain depicted in Fig. 2 ▸. Fig. 5 ▸ presents the fingerprint plots showing all inter­molecular contacts (5*a*) and those showing each of the three most significant ones. The H⋯H contacts (5*b*) comprise 69.2% of the total, consistent with the periphery of the mol­ecule being largely hydrogen atoms. The next most important are the O⋯H/H⋯O contacts at 16.7% of the total (5*c*), which appear as a pair of sharp spikes at *d*_e_ + *d*_i_ ≃ 2.3 Å with broad shoulders at *d*_e_ + *d*_i_ ≃ 2.6 Å: the former represent the N1—H1⋯O1^i^ hydrogen bonds (Table 1 ▸) while the latter are attributed to C—H⋯O contacts, which range from 2.66 to 2.73 Å and are considered to be slightly compressed van der Waals contacts rather than significant C—H⋯O hydrogen bonds. The last are the C⋯H/H⋯C contacts (5***d***) appearing as a pair of broad peaks at *d*_e_ + *d*_i_ ≃ 2.8 Å and attributed, in part, to the C—H⋯π(ring) inter­actions listed in Table 1 ▸ and contributing 14.0% to the total. The results of this analysis show a strong, 1-D supra­molecular component to the crystal but relatively weak inter­actions in the other two directions.

## 6. Synthesis and crystallization

**Figure 6:**
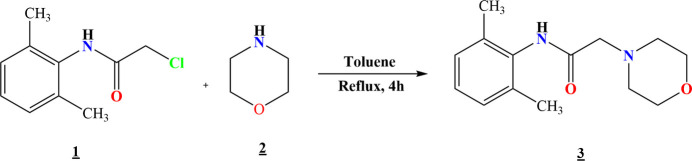
Reaction scheme for the formation of the title compound **3**.

The reaction sequence for preparing **3** is shown in Fig. 6 ▸. 2-Chloro-*N*-(2,6-di­methyl­phen­yl)acetamide, **1**, was synthesized according to the procedure described in the literature (Missioui *et al.*, 2022*b* ▸). Next, 1.2 mmol of morpholine, **2**, were mixed with 1 mmol of 2-chloro-*N*-(4-nitro­phen­yl)acetamide and refluxed in toluene for 4 h. Upon completion of the reaction, toluene was removed by liquid–liquid extraction, and the aqueous phase was subsequently acidified with hydro­chloric acid to adjust its pH to about 4, prompting the precipitation of **3**. The precipitate was filtered off, dried, and recrystallized from ethanol solution, yielding white crystals.

Yield = 45%, color: white, m.p. = 399–401 K. **FT–IR** (ATR, cm^−1^) : 3228 (N—H amide), 2956 (C—H aliphatic), 1661(C=O). **^1^H NMR** (500 MHz, DMSO-*d*_6_) δ(ppm): 2.10 (*s*, 6 H, CH_3_), 2.5 (*t*, 4 H, N—CH_2_—C), 3.09 (*s*, 2 H, CH_2_ amide), 3.62 (*t*, 4 H, O—CH_2_—C), 7.02–7.04 (*m*, 3 H, H_ar_), and 9.20 (*s*, 1 H, NH amide). **^13^C NMR** (125 MHz, DMSO-*d*_6_) δ(ppm): 18.76 (CH_3_), 53.97 (N—CH_2_—C), 62.15 (N—CH_2_—C—O), 66.62 (O—CH_2_—C), 126.94, 128.20, 135.59, 135.74 (C_a_r), and 168.34 (C=O). **HRMS (ESI):** calculated for C_14_H_20_N_2_O_2_ [*M* + H]^+^ 249.1525; found 249.15874.

## 7. Refinement

Crystal data, data collection and structure refinement details are summarized in Table 3 ▸. The N—H H atom was refined freely. C-bound H atoms were positioned with idealized geometry (C—H = 0.93–0.97 Å) and refined using a riding model with *U*_iso_(H) = 1.2 or 1.5*U*_eq_(C).

## Supplementary Material

Crystal structure: contains datablock(s) I. DOI: 10.1107/S2056989026006043/ex2100sup1.cif

Structure factors: contains datablock(s) I. DOI: 10.1107/S2056989026006043/ex2100Isup2.hkl

Supporting information file. DOI: 10.1107/S2056989026006043/ex2100Isup3.cml

CCDC reference: 2560706

Additional supporting information:  crystallographic information; 3D view; checkCIF report

## Figures and Tables

**Table 1:** Hydrogen-bond geometry (Å, °) *Cg*2 is the centroid of the C1–C6 ring.

*D*—H⋯*A*	*D*—H	H⋯*A*	*D*⋯*A*	*D*—H⋯*A*
N1—H1⋯N2	0.88 (2)	2.278 (18)	2.738 (2)	112.5 (16)
N1—H1⋯O1^i^	0.88 (2)	2.34 (2)	3.0798 (18)	141.3 (16)
C10—H10*B*⋯*Cg*2^ii^	0.97	2.94	3.782 (2)	146

Symmetry codes: (i) 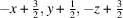; (ii) 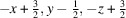.

**Table 2:** Database Survey

REFCODE	*R*	Component 2	Dihedral angle (°)	Reference
BIDVET	NEt_2_	2-*i*-propyl-5-methyl­cyclo­hexa­nol	85.4 (13)	Ma *et al.* (2023 ▸)
BIDVET01	NEt_2_	2-*i*-propyl-5-methyl­cyclo­hexa­nol	85.6 (14)	Ma *et al.* (2023 ▸)
BIDVET02	NEt_2_	2-*i*-propyl-5-methyl­cyclo­hexa­nol	84.4 (6)	Ma *et al.* (2023 ▸)
DALJIN	NEt_2_	nona­nedioic acid	58.90 (15), 65.00 (15)	Zotova *et al.* (2021 ▸)
LIDCAN10	NEt_2_	–	82.0, 77.8	Hanson & Banner (1974 ▸)
LIDCAN11	NEt_2_	–	76.0 (6), 75.6 (6)	Bambagiotti-Alberti *et al.* (2007 ▸)
LIDCAN12	NEt_2_	–	79.79 (15), 72.74 (10), 68.41 (16), 78.93 (16)	Gryl (2015 ▸)
SEQRAJ	NEt_2_	2-*i*-propyl-5-methyl­cyclo­hexa­nol	76.05 (18)	Corvis *et al.* (2010 ▸)
TURNOW	NEt_2_	1,3,5-tri­hydroxy­benzene	71.24 (8)	Magaña-Vergara *et al.* (2018 ▸)
WEDWUA	NEt_2_	1,4-di­bromo-2,3,5,6-tetra­fluoro­benzene	78.42 (17)	Choquesillo-Laza­rte *et al.* (2017 ▸)
WEDXAH	NEt_2_	1,4-di­iodo-2,3,5,6-tetra­fluoro­benzene	86.4 (3)	Choquesillo-Laza­rte *et al.* (2017 ▸)
GENRAT	pyrrolidin-2-one-1-yl	–	66.49 (15)	Wang *et al.* (2006*b* ▸)
KAJSIB	pyrrolidin-2-one-1-yl	2-hy­droxy-2-phenyl­acetic acid	60.87 (16)	Buol *et al.* (2020*a* ▸)
KAJSIB01	pyrrolidin-2-one-1-yl	2-hy­droxy-2-phenyl­acetic acid	60.84 (14)	Buol *et al.* (2020*a* ▸)
OYUWOX	pyrrolidin-2-one-1-yl	2-phenyl­succinic acid	61.23 (18)	Buol *et al.* (2020*a* ▸)
OYUWUD01	pyrrolidin-2-one-1-yl	5-nitro­isophthalic acid	71.59 (13)	Buol *et al.* (2020*a* ▸)
OYUXAK	pyrrolidin-2-one-1-yl	4-hy­droxy­benzoic acid	64.89 (12)	Buol *et al.* (2020*b* ▸)
OYUXIS	pyrrolidin-2-one-1-yl	5-cyano­isophthalic acid	68.75 (19)	Buol *et al.* (2020*b* ▸)
OYUXOY	pyrrolidin-2-one-1-yl	2-benzoyl­benzoic acid	60.03 (17)	Buol *et al.* (2020*b* ▸)
OYUXUE	pyrrolidin-2-one-1-yl	2-hy­droxy-3-phenyl­propanoic acid	60.4 (2)	Buol *et al.* (2020*b* ▸)
OYUYAL	pyrrolidin-2-one-1-yl	2-phenyl­butyric acid	61.6 (8)	Buol *et al.* (2020*b* ▸)
OYUYEP	pyrrolidin-2-one-1-yl	5-hy­droxy­isophthalic acid	74.91 (9), 78.08 (9)	Buol *et al.* (2020*a* ▸)
OYUYIT	pyrrolidin-2-one-1-yl	2-hy­droxy­propane-1,2,3-tri­carb­oxy­lic acid	69 (9)	Buol *et al.* (2020*a* ▸)
OYUYIT01	pyrrolidin-2-one-1-yl	2-hy­droxy­propane-1,2,3-tri­carb­oxy­lic acid	69.7 (9), 69.0 (9), 67.7 (7), 70.5 (8)	Buol *et al.* (2020*b* ▸)
OYUZAM	pyrrolidin-2-one-1-yl	oxalic acid	69.6 (6), 67.0 (7), 68.9 (8), 77.0 (8)	Buol *et al.* (2020*a* ▸)
OYUZOA	pyrrolidin-2-one-1-yl	3,4,5-tri­hydroxy­benzoic acid	70.4 (7), 72.1 (9)	Buol *et al.* (2020*b*)
ACEZAK	4-*R*′-piperazin-1-yl^*a*^	–	64.23 (19)	Wang *et al.* (2004 ▸)
CINBEK	4-*R*′-piperazin-1-yl^*b*^	–	57.2 (2)	Silva *et al.* (2023 ▸)
JAYSAE	4-*R*′-piperazin-1-yl^*c*^	–	67.73 (16)	Wang *et al.* (2005*b* ▸)
LIPFAZ	4-*R*′’-piperazin-1-yl^*d*^	–	73.3 (4)	Germain *et al.* (1977 ▸)
MAPKIY	4-*R*′-piperazin-1-yl^*e*^	–	63.86 (17)	Wang *et al.* (2005*a* ▸)
SENCAQ	4-*R*′-piperazin-1-yl^*f*^	–	65.5 (6)	Wang *et al.* (2006*a* ▸)
SEJLOM	3-hy­droxy-3-meth­oxy­methyl-2-oxoindolin-1-yl	–	72.76 (8)	Nchioua *et al.* (2022 ▸)
VAVGIM	4-(2,6-di­methyl­phen­yl)-3,5-dioxopiperazin-1-yl	–	73.98 (15)	Heim *et al.* (2021 ▸)
YOTROO	N(CH_2_COOH)_2_	–	60.88 (18)	Ribár *et al.* (1995 ▸)
YOTRUU	N(CH_2_COOH)[CH_2_C(O)OMe]	–	74.5 (4)	Ribár *et al.* (1995 ▸)

Notes: (*a*) *R*′ = 3-(4-nitro­phen­yl)-1,2,4-oxa­diazol-5-ylmethyl; (*b*) *R*′ = 2-hy­droxy-3-(2-meth­oxy­phen­oxy)propyl; (*c*) *R*′ = 3-(3-meth­oxy­phen­yl)-1,2,4-oxa­diazol-5-ylmethyl; (*d*) *R*′ = 4,4-bis­(4-fluoro­phen­yl)butyl; (*e*) *R*′ = 3-(2-chloro­phen­yl)-1,2,4-oxa­diazol-5-ylmethyl; (*f*) *R*′ = 3-(4-bromo­phen­yl)-1,2,4-oxa­diazol-5-ylmethyl.

**Table 3:** Experimental details

Crystal data	
Chemical formula	C_14_H_20_N_2_O_2_
*M*_r_	248.32
Crystal system, space group	Monoclinic, *P*2_1_/*n*
Temperature (K)	293
*a*, *b*, *c* (Å)	12.2417 (9), 10.4498 (4), 12.2866 (9)
β (°)	118.704 (9)
*V* (Å^3^)	1378.59 (18)
*Z*	4
Radiation type	Cu *K*α
μ (mm^−1^)	0.65
Crystal size (mm)	0.49 × 0.23 × 0.14

Data collection	
Diffractometer	SuperNova, Dual, Cu at home/near, Atlas
Absorption correction	Gaussian (*CrysAlis PRO*; Rigaku OD, 2024 ▸)
*T*_min_, *T*_max_	0.326, 1.000
No. of measured, independent and observed [*I* > 2σ(*I*)] reflections	9760, 2709, 1991
*R*_int_	0.028
(sin θ/λ)_max_ (Å^−1^)	0.620

Refinement	
*R*[*F*^2^ > 2σ(*F*^2^)], *wR*(*F*^2^), *S*	0.046, 0.142, 1.04
No. of reflections	2709
No. of parameters	169
No. of restraints	1
H-atom treatment	H atoms treated by a mixture of independent and constrained refinement
Δρ_max_, Δρ_min_ (e Å^−3^)	0.15, −0.18

Computer programs: *CrysAlis PRO* (Rigaku OD, 2024 ▸), *SHELXT* (Sheldrick, 2015*a* ▸), *SHELXL* (Sheldrick, 2015*b* ▸) and , *DIAMOND* (Brandenburg & Putz, 2012 ▸).

## supplementary crystallographic information

### N-(2,6-Dimethylphenyl)-2-(morpholin-4-yl)acetamide Crystal data

**Table:**

C_14_H_20_N_2_O_2_	*F*(000) = 536
*M**_r_* = 248.32	*D*_x_ = 1.196 Mg m^−^^3^
Monoclinic, *P*2_1_/*n*	Cu *K*α radiation, λ = 1.54184 Å
*a* = 12.2417 (9) Å	Cell parameters from 3200 reflections
*b* = 10.4498 (4) Å	θ = 4.2–72.3°
*c* = 12.2866 (9) Å	µ = 0.65 mm^−^^1^
β = 118.704 (9)°	*T* = 293 K
*V* = 1378.59 (18) Å^3^	Block, colourless
*Z* = 4	0.49 × 0.23 × 0.14 mm

### N-(2,6-Dimethylphenyl)-2-(morpholin-4-yl)acetamide Data collection

**Table:**

SuperNova, Dual, Cu at home/near, Atlas diffractometer	1991 reflections with *I* > 2σ(*I*)
Detector resolution: 10.5082 pixels mm^-1^	*R*_int_ = 0.028
ω scans	θ_max_ = 73.0°, θ_min_ = 4.2°
Absorption correction: gaussian (CrysAlisPro; Rigaku OD, 2024)	*h* = −13→15
*T*_min_ = 0.326, *T*_max_ = 1.000	*k* = −12→12
9760 measured reflections	*l* = −15→14
2709 independent reflections	

### N-(2,6-Dimethylphenyl)-2-(morpholin-4-yl)acetamide Refinement

**Table:**

Refinement on *F*^2^	1 restraint
Least-squares matrix: full	Hydrogen site location: mixed
*R*[*F*^2^ > 2σ(*F*^2^)] = 0.046	H atoms treated by a mixture of independent and constrained refinement
*wR*(*F*^2^) = 0.142	*w* = 1/[σ^2^(*F*_o_^2^) + (0.0713*P*)^2^ + 0.2035*P*] where *P* = (*F*_o_^2^ + 2*F*_c_^2^)/3
*S* = 1.03	(Δ/σ)_max_ < 0.001
2709 reflections	Δρ_max_ = 0.15 e Å^−^^3^
169 parameters	Δρ_min_ = −0.18 e Å^−^^3^

### N-(2,6-Dimethylphenyl)-2-(morpholin-4-yl)acetamide Special details

**Table:**

Geometry. All esds (except the esd in the dihedral angle between two l.s. planes) are estimated using the full covariance matrix. The cell esds are taken into account individually in the estimation of esds in distances, angles and torsion angles; correlations between esds in cell parameters are only used when they are defined by crystal symmetry. An approximate (isotropic) treatment of cell esds is used for estimating esds involving l.s. planes.
Refinement. Single-crystal X-ray diffraction data were collected on an Agilent SuperNova Dual Atlas diffractometer, equipped with a mirror monochromator and using Mo radiation. The data were processed using CrysAlisPro (Rigaku OD, 2024) and the crystal structures were solved using SHELXT(Sheldrick, 2015a) and refined using SHELXL(Sheldrick, 2015b). Non-hydrogen atoms were refined with anisotropic displacement parameters.

### N-(2,6-Dimethylphenyl)-2-(morpholin-4-yl)acetamide Fractional atomic coordinates and isotropic or equivalent isotropic displacement parameters (Å^2^)

**Table:**

	*x*	*y*	*z*	*U*_iso_*/*U*_eq_	
C1	0.59743 (15)	0.60953 (13)	0.71939 (15)	0.0484 (4)	
C2	0.51238 (15)	0.65227 (14)	0.59945 (16)	0.0515 (4)	
C3	0.38984 (18)	0.67279 (17)	0.5722 (2)	0.0661 (5)	
H3	0.332387	0.701082	0.493473	0.079*	
C4	0.3519 (2)	0.6523 (2)	0.6586 (2)	0.0799 (6)	
H4	0.269123	0.665369	0.638096	0.096*	
C5	0.4366 (2)	0.6123 (2)	0.7762 (2)	0.0776 (6)	
H5	0.409988	0.599209	0.834640	0.093*	
C6	0.56176 (18)	0.59086 (16)	0.80978 (18)	0.0601 (4)	
C7	0.55179 (17)	0.67607 (17)	0.50351 (16)	0.0627 (5)	
H7A	0.615717	0.740284	0.532914	0.094*	
H7B	0.481579	0.704990	0.428263	0.094*	
H7C	0.583419	0.598230	0.487688	0.094*	
C8	0.6532 (2)	0.5506 (2)	0.94016 (19)	0.0789 (6)	
H8A	0.623068	0.576806	0.995803	0.118*	
H8B	0.732437	0.590077	0.964372	0.118*	
H8C	0.662476	0.459214	0.943309	0.118*	
C9	0.77763 (16)	0.47292 (15)	0.76710 (16)	0.0552 (4)	
C10	0.90876 (18)	0.47159 (18)	0.7846 (2)	0.0701 (5)	
H10A	0.967251	0.474147	0.872751	0.084*	
H10B	0.921820	0.391831	0.752147	0.084*	
C11	1.06707 (17)	0.6107 (2)	0.77925 (19)	0.0723 (6)	
H11A	1.113068	0.540388	0.768972	0.087*	
H11B	1.100787	0.626798	0.867333	0.087*	
C12	1.0799 (2)	0.7277 (3)	0.7164 (3)	0.0927 (7)	
H12A	1.033815	0.797529	0.727217	0.111*	
H12B	1.166922	0.752477	0.754614	0.111*	
C13	0.9087 (2)	0.6687 (3)	0.5317 (2)	0.0856 (7)	
H13A	0.879929	0.650423	0.444758	0.103*	
H13B	0.859282	0.738908	0.536367	0.103*	
C14	0.88885 (18)	0.55288 (19)	0.59218 (18)	0.0681 (5)	
H14A	0.800777	0.532485	0.552940	0.082*	
H14B	0.932346	0.480240	0.581836	0.082*	
N1	0.72330 (13)	0.58889 (13)	0.74639 (13)	0.0509 (3)	
N2	0.93530 (13)	0.57730 (13)	0.72435 (13)	0.0560 (4)	
O1	0.72824 (12)	0.37440 (11)	0.77594 (13)	0.0708 (4)	
O2	1.03491 (16)	0.70609 (19)	0.58815 (17)	0.0980 (5)	
H1	0.7656 (17)	0.651 (2)	0.7350 (17)	0.063 (5)*	

### N-(2,6-Dimethylphenyl)-2-(morpholin-4-yl)acetamide Atomic displacement parameters (Å^2^)

**Table:**

	*U*^11^	*U*^22^	*U*^33^	*U*^12^	*U*^13^	*U*^23^
C1	0.0522 (9)	0.0332 (7)	0.0626 (10)	−0.0040 (6)	0.0298 (8)	−0.0032 (6)
C2	0.0541 (9)	0.0345 (7)	0.0616 (10)	−0.0016 (6)	0.0244 (8)	−0.0046 (6)
C3	0.0557 (11)	0.0564 (10)	0.0773 (12)	0.0046 (8)	0.0247 (9)	−0.0071 (9)
C4	0.0616 (12)	0.0824 (14)	0.0993 (16)	0.0035 (10)	0.0415 (12)	−0.0113 (12)
C5	0.0845 (14)	0.0762 (13)	0.0971 (15)	−0.0076 (11)	0.0636 (13)	−0.0083 (11)
C6	0.0716 (11)	0.0480 (8)	0.0692 (11)	−0.0065 (8)	0.0405 (9)	−0.0021 (7)
C7	0.0663 (11)	0.0556 (9)	0.0587 (10)	−0.0013 (8)	0.0240 (9)	0.0031 (8)
C8	0.1016 (16)	0.0738 (13)	0.0672 (12)	−0.0096 (11)	0.0454 (12)	0.0046 (10)
C9	0.0610 (10)	0.0412 (8)	0.0593 (9)	0.0059 (7)	0.0256 (8)	0.0059 (7)
C10	0.0655 (11)	0.0585 (10)	0.0839 (13)	0.0172 (9)	0.0340 (10)	0.0170 (9)
C11	0.0520 (10)	0.0900 (14)	0.0732 (12)	0.0018 (10)	0.0286 (9)	−0.0206 (10)
C12	0.0801 (15)	0.1038 (18)	0.1115 (19)	−0.0305 (13)	0.0599 (14)	−0.0280 (15)
C13	0.0780 (15)	0.1089 (18)	0.0800 (14)	0.0046 (12)	0.0459 (12)	0.0102 (12)
C14	0.0577 (11)	0.0731 (12)	0.0684 (11)	0.0027 (9)	0.0261 (9)	−0.0114 (9)
N1	0.0518 (8)	0.0385 (6)	0.0625 (8)	0.0000 (6)	0.0274 (7)	0.0047 (6)
N2	0.0500 (8)	0.0539 (8)	0.0638 (9)	0.0054 (6)	0.0270 (7)	−0.0005 (6)
O1	0.0787 (9)	0.0401 (6)	0.0902 (9)	0.0026 (6)	0.0376 (7)	0.0090 (6)
O2	0.0848 (11)	0.1313 (15)	0.1008 (12)	−0.0166 (10)	0.0629 (10)	−0.0041 (10)

### N-(2,6-Dimethylphenyl)-2-(morpholin-4-yl)acetamide Geometric parameters (Å, º)

**Table:**

C1—C6	1.388 (2)	C9—C10	1.514 (3)
C1—C2	1.408 (2)	C10—N2	1.451 (2)
C1—N1	1.428 (2)	C10—H10A	0.9700
C2—C3	1.388 (2)	C10—H10B	0.9700
C2—C7	1.493 (2)	C11—N2	1.461 (2)
C3—C4	1.363 (3)	C11—C12	1.495 (3)
C3—H3	0.9300	C11—H11A	0.9700
C4—C5	1.378 (3)	C11—H11B	0.9700
C4—H4	0.9300	C12—O2	1.415 (3)
C5—C6	1.400 (3)	C12—H12A	0.9700
C5—H5	0.9300	C12—H12B	0.9700
C6—C8	1.508 (3)	C13—O2	1.412 (3)
C7—H7A	0.9600	C13—C14	1.499 (3)
C7—H7B	0.9600	C13—H13A	0.9700
C7—H7C	0.9600	C13—H13B	0.9700
C8—H8A	0.9600	C14—N2	1.462 (2)
C8—H8B	0.9600	C14—H14A	0.9700
C8—H8C	0.9600	C14—H14B	0.9700
C9—O1	1.225 (2)	N1—H1	0.88 (2)
C9—N1	1.347 (2)		
			
C6—C1—C2	121.55 (16)	C9—C10—H10A	108.8
C6—C1—N1	120.77 (15)	N2—C10—H10B	108.8
C2—C1—N1	117.65 (15)	C9—C10—H10B	108.8
C3—C2—C1	118.14 (17)	H10A—C10—H10B	107.7
C3—C2—C7	120.37 (16)	N2—C11—C12	108.90 (17)
C1—C2—C7	121.48 (15)	N2—C11—H11A	109.9
C4—C3—C2	121.4 (2)	C12—C11—H11A	109.9
C4—C3—H3	119.3	N2—C11—H11B	109.9
C2—C3—H3	119.3	C12—C11—H11B	109.9
C3—C4—C5	119.9 (2)	H11A—C11—H11B	108.3
C3—C4—H4	120.1	O2—C12—C11	111.30 (19)
C5—C4—H4	120.1	O2—C12—H12A	109.4
C4—C5—C6	121.5 (2)	C11—C12—H12A	109.4
C4—C5—H5	119.2	O2—C12—H12B	109.4
C6—C5—H5	119.2	C11—C12—H12B	109.4
C1—C6—C5	117.51 (18)	H12A—C12—H12B	108.0
C1—C6—C8	122.04 (17)	O2—C13—C14	112.33 (19)
C5—C6—C8	120.45 (18)	O2—C13—H13A	109.1
C2—C7—H7A	109.5	C14—C13—H13A	109.1
C2—C7—H7B	109.5	O2—C13—H13B	109.1
H7A—C7—H7B	109.5	C14—C13—H13B	109.1
C2—C7—H7C	109.5	H13A—C13—H13B	107.9
H7A—C7—H7C	109.5	N2—C14—C13	109.88 (17)
H7B—C7—H7C	109.5	N2—C14—H14A	109.7
C6—C8—H8A	109.5	C13—C14—H14A	109.7
C6—C8—H8B	109.5	N2—C14—H14B	109.7
H8A—C8—H8B	109.5	C13—C14—H14B	109.7
C6—C8—H8C	109.5	H14A—C14—H14B	108.2
H8A—C8—H8C	109.5	C9—N1—C1	124.03 (14)
H8B—C8—H8C	109.5	C9—N1—H1	114.7 (12)
O1—C9—N1	123.59 (16)	C1—N1—H1	120.2 (12)
O1—C9—C10	120.97 (15)	C10—N2—C11	114.49 (15)
N1—C9—C10	115.40 (14)	C10—N2—C14	111.77 (15)
N2—C10—C9	113.74 (14)	C11—N2—C14	107.98 (14)
N2—C10—H10A	108.8	C13—O2—C12	109.88 (16)
			
N1—C9—C10—N2	−26.1 (2)		

### N-(2,6-Dimethylphenyl)-2-(morpholin-4-yl)acetamide Hydrogen-bond geometry (Å, º)

*Cg*2 is the centroid of the C1–C6 ring.

**Table:**

*D*—H···*A*	*D*—H	H···*A*	*D*···*A*	*D*—H···*A*
N1—H1···N2	0.88 (2)	2.278 (18)	2.738 (2)	112.5 (16)
N1—H1···O1^i^	0.88 (2)	2.34 (2)	3.0798 (18)	141.3 (16)
C10—H10*B*···*Cg*2^ii^	0.97	2.94	3.782 (2)	146

Symmetry codes: (i) −*x*+3/2, *y*+1/2, −*z*+3/2; (ii) −*x*+3/2, *y*−1/2, −*z*+3/2.
